# Supplementary material for: Efficacy and Safety of Rechallenge with BRAF/MEK Inhibitors in Advanced Melanoma Patients: A Systematic Review and Meta-Analysis
Source: Cancers (Basel). 2023 Jul 25;15(15):3754. doi: 10.3390/cancers15153754 (PMC10417341; doi:10.3390/cancers15153754)
Supplement: Supplementary file 1 [file cancers-15-03754-s001.zip › Supplementary Materials/Table_S3.docx]

**Table S3.** Full list of excluded studies after a comprehensive analysis.

| # | **Author, year** | **Title** | **Reason for exclusion** |
| --- | --- | --- | --- |
| 1 | Ackerman, 2012 | Outcomes of patients with malignant melanoma treated with immunotherapy prior to or after vemurafenib. | No rechallenge. |
| 2 | Algazi, 2015 | SWOG S1320: a randomized phase II trial of intermittent versus continuous dosing of dabrafenib and trametinib in BRAFV600E/k mutant melanoma | No rechallenge. |
| 3 | Algazi, 2020 | Association of prior immune checkpoint blockade (ICB) with longer progression-free survival (PFS) in patients treated with intermittent versus continuous dabrafenib and trametinib: a post-hoc analysis of S1320 | No rechallenge. |
| 4 | Amin, 2016 | Phase II study of vemurafenib followed by ipilimumab in patients with previously untreated BRAF-mutated metastatic melanoma | No rechallenge. |
| 5 | Arance, 2017 | Safety of vemurafenib in patients with BRAF V600 mutated metastatic melanoma: the Spanish experience | No rechallenge. |
| 6 | Ascierto, 2017 | SECOMBIT (sequential combo immuno and target therapy study): a three arms prospective, randomized phase II study to evaluate the best sequential approach with combo immunotherapy [ipilimumab (I) /nivolumab (N)] and combo target therapy [encorafenib (E)/binimetinib (B)] in patients with metastatic melanoma and BRAF mutation | No rechallenge. |
| 7 | Ascierto, 2022 | Sequencing of Ipilimumab Plus Nivolumab and Encorafenib Plus Binimetinib for Untreated BRAF-Mutated Metastatic Melanoma (SECOMBIT): A Randomized, Three-Arm, Open-Label Phase II Trial | No rechallenge. |
| 8 | Atkins, 2022 | Combination Dabrafenib and Trametinib Versus Combination Nivolumab and Ipilimumab for Patients With Advanced BRAF-Mutant Melanoma: the DREAMseq Trial-ECOG-ACRIN EA6134 | No rechallenge. |
| 9 | Bédouelle, 2022 | Should Targeted Therapy Be Continued in BRAF-Mutant Melanoma Patients after Complete Remission? | No rechallenge. Patients with CR were retreated after discontinuation. |
| 10 | Chen, 2016 | Clinical, Molecular, and Immune Analysis of Dabrafenib-Trametinib Combination Treatment for BRAF Inhibitor–Refractory Metastatic Melanoma: A Phase 2 Clinical Trial | No rechallenge. Most patients were re-introduced to BRAFi/MEKi without an interval. Insufficient data to include subgroups of patients who had an interval. |
| 11 | Desvignes, 2017 | BRAF inhibitor discontinuation and rechallenge in advanced melanoma patients with a complete initial treatment response. | No rechallenge. BRAFi were discontinued in all 11  metastatic melanoma patients with a CR  or with a partial response combined with surgical removal  of the remaining tumours. |
| 12 | Dummer, 2020 | A phase II, multicenter study of encorafenib/binimetinib followed by a rational triple-combination after progression in patients with advanced BRAF V600-mutated melanoma (LOGIC2). | No rechallenge. |
| 13 | Flaherty, 2021 | A study of GSK1120212 compared with chemotherapy in patients with BRAF mutation positive advanced or metastatic melanoma (Linked to: Improved survival with MEK inhibition in BRAF-mutated melanoma) | No rechallenge. |
| 14 | Haist, 2022 | The Role of Treatment Sequencing with Immune-Checkpoint Inhibitors and BRAF/MEK Inhibitors for Response and Survival of Patients with BRAFV600-Mutant Metastatic Melanoma—A Retrospective, Real-World Cohort Study | No rechallenge. |
| 15 | Hassel, 2018 | Progression patterns under BRAF inhibitor treatment and treatment beyond progression in patients with metastatic melanoma | No rechallenge. |
| 16 | Johnson, 2014 | Combined BRAF (Dabrafenib) and MEK Inhibition (Trametinib) in Patients With BRAFV600-Mutant Melanoma Experiencing Progression With Single-Agent BRAF Inhibitor | No rechallenge. Interval to re-introduction of BRAFi/MEKi was not informed. Patients did not receive any treatment between PD and re-exposure to TT. Some patients (arm C) receiving BRAFi alone were crossed over to receive the comobination of BRAFi/MEKi. |
| 17 | Kim, 2012 | Phase II Study of the MEK1/MEK2 Inhibitor Trametinib in Patients With Metastatic BRAF-Mutant Cutaneous Melanoma Previously Treated With or Without a BRAF Inhibitor | No rechallenge. |
| 18 | Pinto, 2022 | Treatment and overall survival among anti-PD-1-exposed advanced melanoma patients with evidence of disease progression | No rechallenge. |
| 19 | Puzanov, 2015 | Long-term outcome in BRAFV600E melanoma patients treated with vemurafenib: Patterns of disease progression and clinical management of limited progression | No rechallenge. |
| 20 | Scholtens, 2015 | Vemurafenib for BRAF V600 mutated advanced melanoma: Results of treatment beyond progression | No rechallenge. |
| 21 | Schreuer, 2016 | COMBI-rechallenge: a phase II clinical trial on dabrafenib plus trametinib in BRAFV600-mutant melanoma patients who previously experienced progression on BRAF(+MEK)-inhibition | Overlapping populations with an included study (Schreuer, 2017). |
| 22 | Sponsor: F. HOFFMANN-LA ROCHE LTD. | A PHASE IV, POSTMARKETING, OPEN-LABEL, EXTENSION (ROLLOVER) STUDY OF VEMURAFENIB IN PATIENTS WITH BRAFV600 MUTATION−POSITIVE MALIGNANCIES PREVIOUSLY ENROLLED IN AN ANTECEDENT VEMURAFENIB PROTOCOL | No rechallenge. Study protocol. No results available. |
| 23 | Sponsor: INTERGRUPPO MELANOMA ITALIANO | To evaluate the efficacy beyond progression of vemurafenib combined with cobimetinib associated with local treatment compared to second-line treatment in patients with BRAFV600 mutation-positive metastatic melanoma in focal progression with first-line combined vemurafenib and cobimetinib | No rechallenge. Study protocol. No results available. |
| 24 | Sponsor: Novartis Pharma AG | A randomized, open-label, multi-arm, two-part, phase II study to assess the efficacy and safety of multiple LXH254 combinations in patients with previously treated unresectable or metastatic BRAFV600 or NRAS mutant melanoma | No rechallenge. Study protocol. No results available. |
| 25 | Stege, 2021 | Discontinuation of BRAF/MEK-Directed Targeted Therapy after Complete Remission of Metastatic Melanoma—A Retrospective Multicenter ADOReg Study | No rechallenge. This study just analyses patients with CR who discontinued treatment and received other treatments. Some patients were re-introduced to BRAFi/MEKi without an interval. Insufficient data to include subgroups of patients who had an interval. |
| 26 | Weichenthal, 2019 | Salvage therapy after failure from anti-PD-1 single agent treatment: A Study by the German ADOReg melanoma registry. | Insufficient data. |
| 27 | Wen, 2020 | A real-world study of vemurafenib plus anti-PD-1 antibody in Chinese patients with advanced BRAF V600-mutant melanoma | No rechallenge. |

CR: complete response. PD: progressed disease. TT: targeted therapy. BRAFi: BRAF inhibitor. MEKi: MEK inhibitor.
