# Supplementary material for: Efficacy and Safety of Rechallenge with BRAF/MEK Inhibitors in Advanced Melanoma Patients: A Systematic Review and Meta-Analysis
Source: Cancers (Basel). 2023 Jul 25;15(15):3754. doi: 10.3390/cancers15153754 (PMC10417341; doi:10.3390/cancers15153754)
Supplement: Supplementary file 1 [file cancers-15-03754-s001.zip › Supplementary Materials/Figure_S3.pdf]

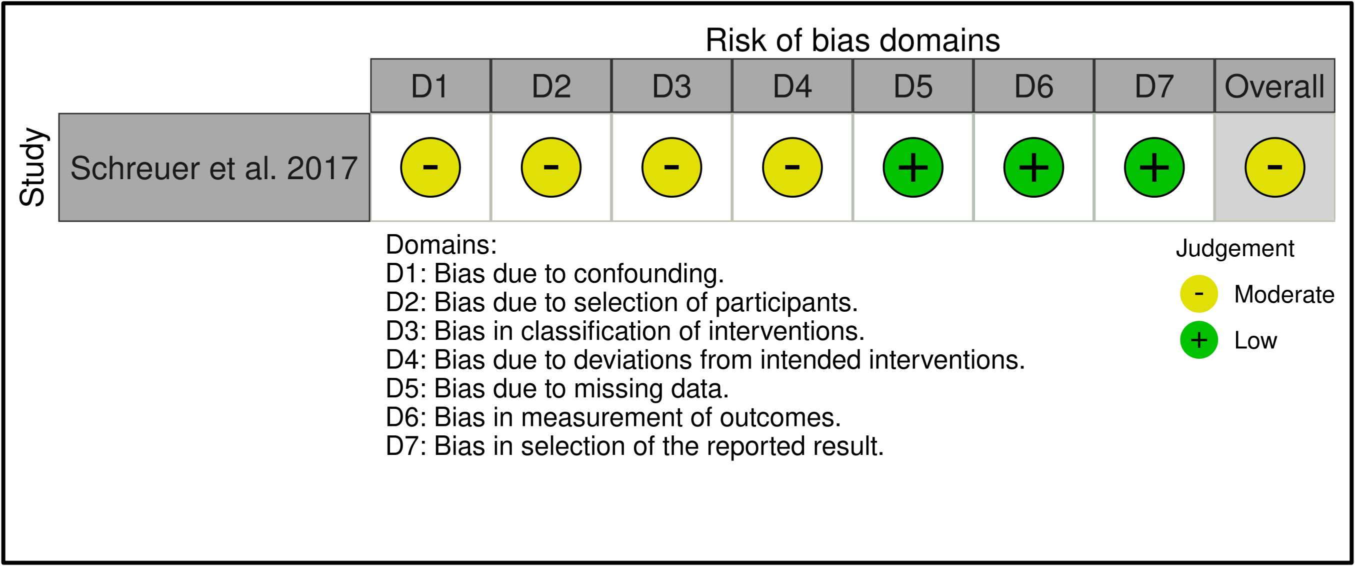
Risk of bias domains
D1
D2
D3
D4
D5
D6
D7
Overall
Study
Schreuer et al. 2017
-
-
-
-
+
+
+
-
Domains:
D1: Bias due to confounding.
D2: Bias due to selection of participants.
D3: Bias in classification of interventions.
D4: Bias due to deviations from intended interventions.
D5: Bias due to missing data.
D6: Bias in measurement of outcomes.
D7: Bias in selection of the reported result.
Judgement
- Moderate
+ Low
