# Supplementary material for: Efficacy and Safety of Rechallenge with BRAF/MEK Inhibitors in Advanced Melanoma Patients: A Systematic Review and Meta-Analysis
Source: Cancers (Basel). 2023 Jul 25;15(15):3754. doi: 10.3390/cancers15153754 (PMC10417341; doi:10.3390/cancers15153754)
Supplement: Supplementary file 1 [file cancers-15-03754-s001.zip › Supplementary Materials/Table_S4.docx]

­­­­**Table S4.** Description of best response during first targeted therapy (TT) exposure (in the 1st/2^nd^ line of treatment) and the best response with TT rechallenge in each study.

| Study ID | N | Best Response During 1^st^/2^nd^ line TT IT with ICI | | | | Best Response to Rechallenge | | | |
| --- | --- | --- | --- | --- | --- | --- | --- | --- | --- |
|  |  | CR | PR | SD | PD | CR | PR | SD | PD |
| Atkinson 2020 | 90 | 18 | 37 | 15 | 12 | 5 | 24 | 17 | 44 |
| Cybulska-Stopa 2020 | 51 | 6 | 30 | 11 | 4 | 0 | 14 | 18 | 16 |
| Persa 2021 | 48 | 3 | 39 | 3 | 3 | 0 | 16 | 13 | 16 |
| Roux 2015 | 10 | 0 | 8 | 0 | 2 | 0 | 5 | 3 | 1 |
| Schreuer 2017 | 25 | 4 | 19 | 1 | 1 | 0 | 8 | 10 | 7 |
| Tietze 2018 | 60 | 7 | 35 | 12 | 4 | 5 | 12 | 17 | 19 |
| Valpione 2017 | 116 | 14 | 52 | 30 | 12 | 3 | 46 | 28 | 36 |

ID: identification. N: number of patients. CR: complete response. PR: partial response. SD: stable disease. PD: progressive disease.
