# Supplementary material for: Efficacy and Safety of Rechallenge with BRAF/MEK Inhibitors in Advanced Melanoma Patients: A Systematic Review and Meta-Analysis
Source: Cancers (Basel). 2023 Jul 25;15(15):3754. doi: 10.3390/cancers15153754 (PMC10417341; doi:10.3390/cancers15153754)
Supplement: Supplementary file 1 [file cancers-15-03754-s001.zip › Supplementary Materials/Figure_S2.pdf]

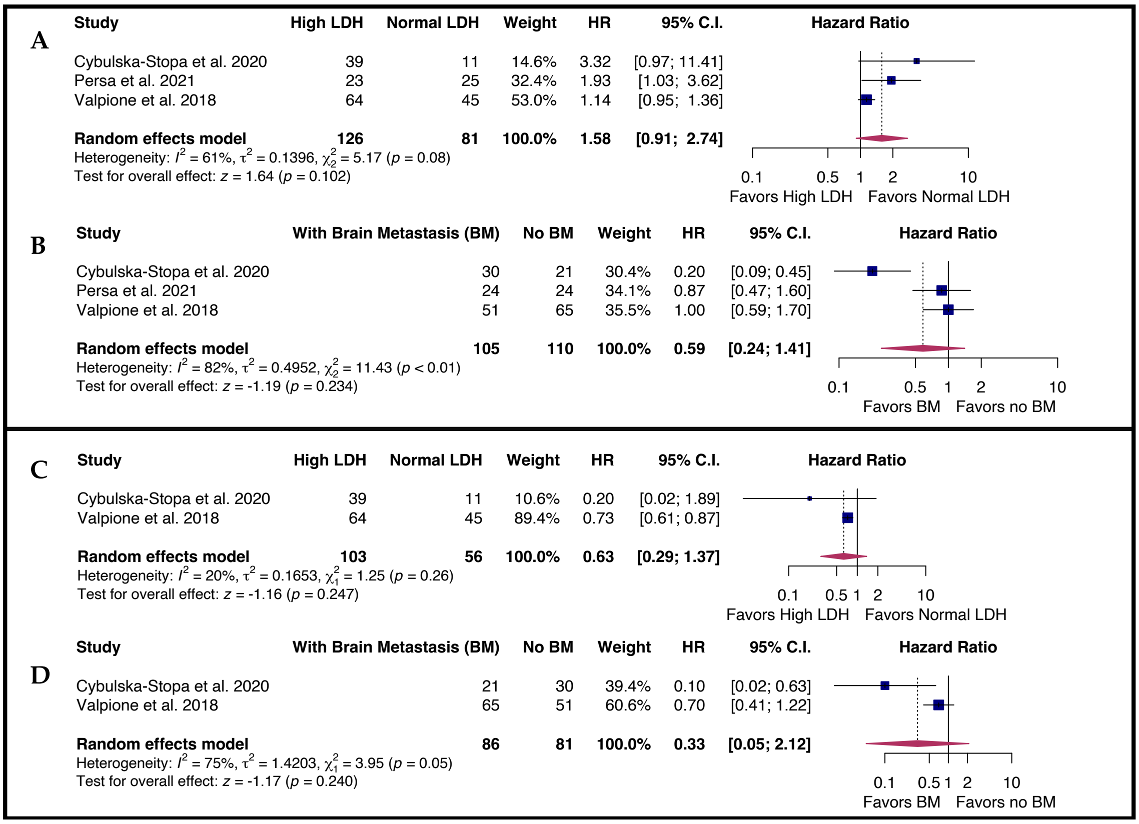

Figure S2. Prognostic factors associated with OS (A and B) and PFS (C and D). Interpretation of each forest plot:

A: Patients with elevated LDH levels had a 58% higher risk of death than those with normal LDH, which did not reach statistical significance (HR = 1.58, p = 0.102).

B: The absence of brain metastasis (BM) non-significantly decreased the risk of death by 41% compared with the presence of BM (HR = 0.59, p = 0.234).

C: Normal LDH was associated with a non-significant reduction of disease progression or death compared to high LDH levels (HR = 0.63, p = 0.247).

D: Patients without BM had a non-significant decrease of 66% in the risk of progression or death compared to patients with BM (HR = 0.33, p = 0.240).
