# Supplementary material for: Efficacy and Safety of Rechallenge with BRAF/MEK Inhibitors in Advanced Melanoma Patients: A Systematic Review and Meta-Analysis
Source: Cancers (Basel). 2023 Jul 25;15(15):3754. doi: 10.3390/cancers15153754 (PMC10417341; doi:10.3390/cancers15153754)
Supplement: Supplementary file 1 [file cancers-15-03754-s001.zip › Supplementary Materials/Table_S5.docx]

**Table S5**. Individual study appraisal for assessing the quality of nonrandomized studies in meta-analyses using The Newcastle-Ottawa Scale (NOS).

| **Study ID** | **Items** | | | | | | | | |
| --- | --- | --- | --- | --- | --- | --- | --- | --- | --- |
|  | **Selection** | | | | **Comparability** | **Exposure** | | |  |
|  | **1** | **2** | **3** | **4** | **1** | **1** | **2** | **3** |  |
| Atkinson et al. 2020 | 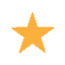 | - | 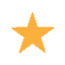 | 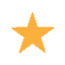 | 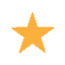 | 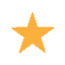 | 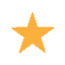 | 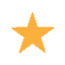 | **7/9** |
| Cybulska-Stopa et al. 2020 | 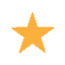 | - | 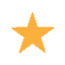 | 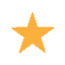 | 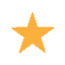 | 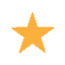 | 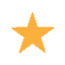 | 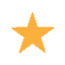 | **7/9** |
| Persa et al. 2021 | 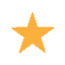 | - | 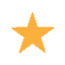 | 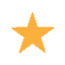 | 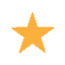 | 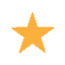 | 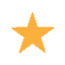 | 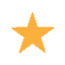 | **7/9** |
| Roux et al. 2015 | 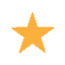 | - | 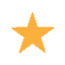 | 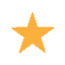 | - | 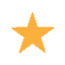 | 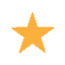 | 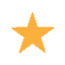 | **6/9** |
| Tietze et al. 2018 | 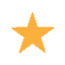 | - | 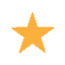 | 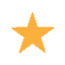 | 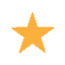 | 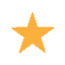 | 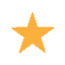 | 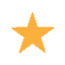 | **7/9** |
| Valpione et al. 2018 | 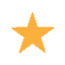 | - | 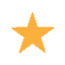 | 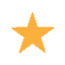 | 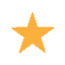 | 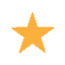 | 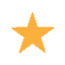 | 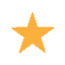 | **7/9** |
