# Supplementary figures and images for: Efficacy and Safety of Rechallenge with BRAF/MEK Inhibitors in Advanced Melanoma Patients: A Systematic Review and Meta-Analysis
Source: Cancers (Basel). 2023 Jul 25;15(15):3754. doi: 10.3390/cancers15153754 (PMC10417341; doi:10.3390/cancers15153754)

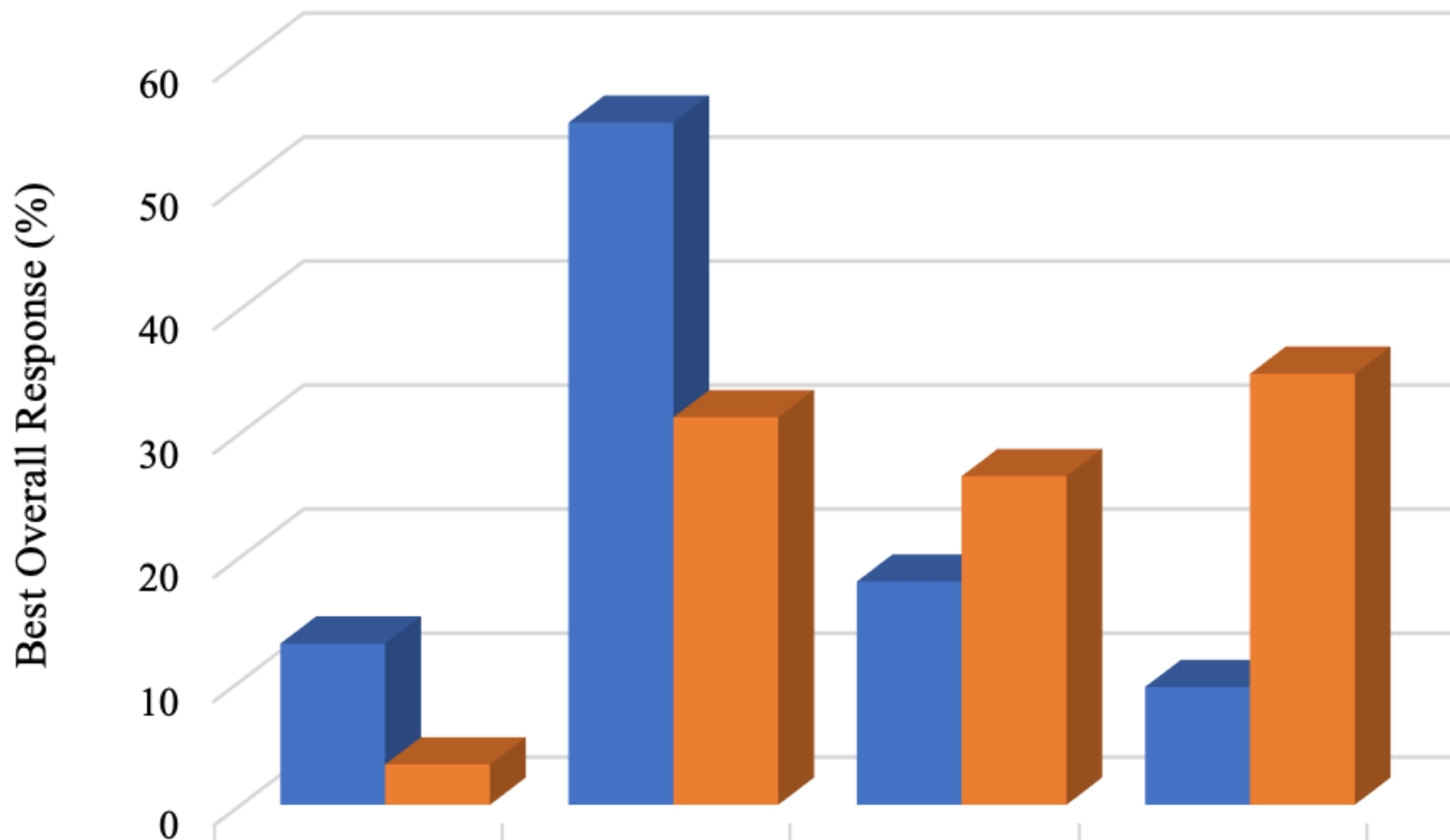

|                   |      |       |      |       |
|-------------------|------|-------|------|-------|
| ■ 1st TT exposure | CR   | PR    | SD   | PD    |
| ■ TT Rechallenge  | 13   | 55    | 18   | 9.5   |
|                   | 3.25 | 31.25 | 26.5 | 34.75 |

Supplement: Supplementary file 1 [file cancers-15-03754-s001.zip › Supplementary Materials/Figure_S1.pdf]

A

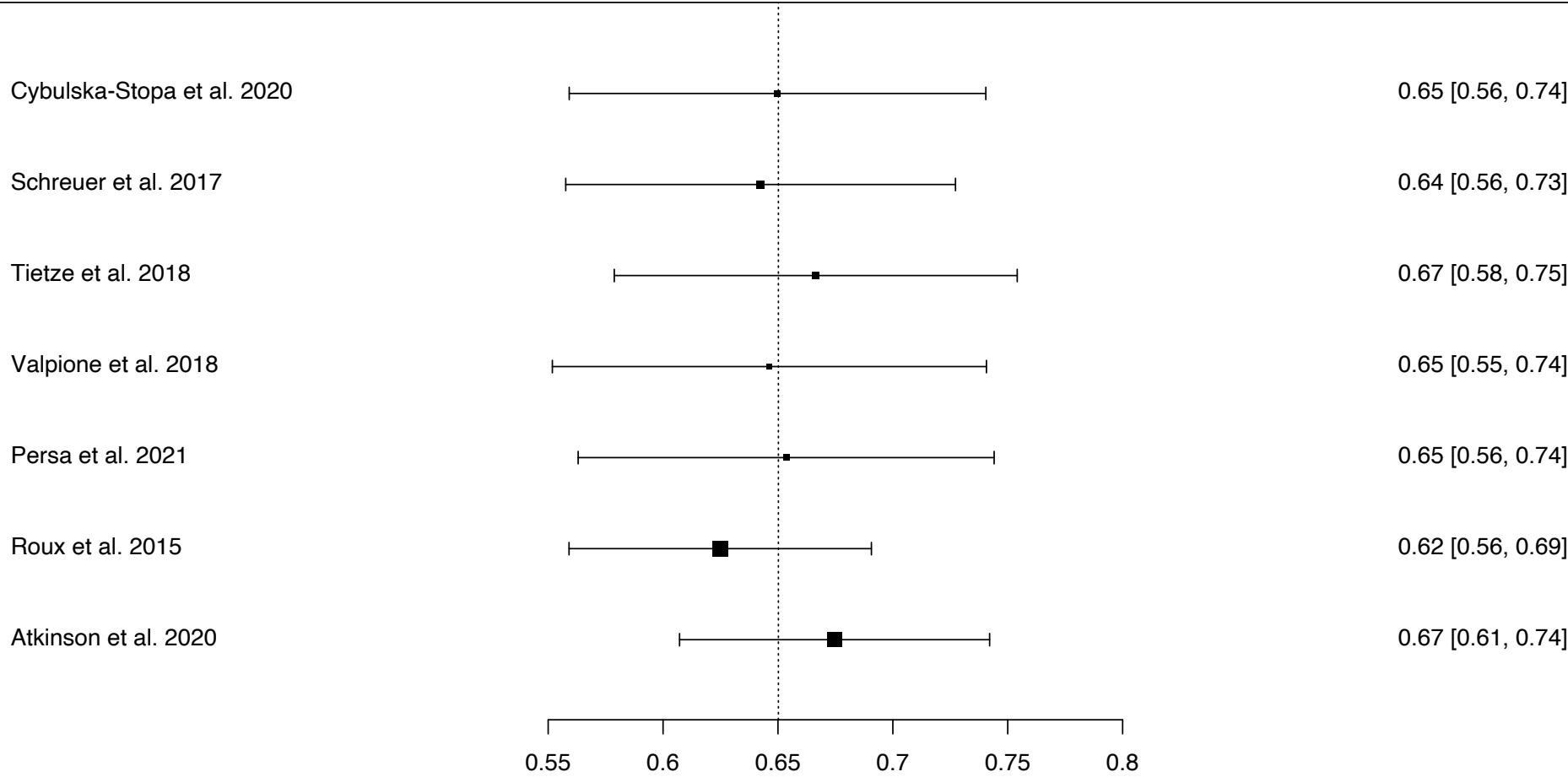

B

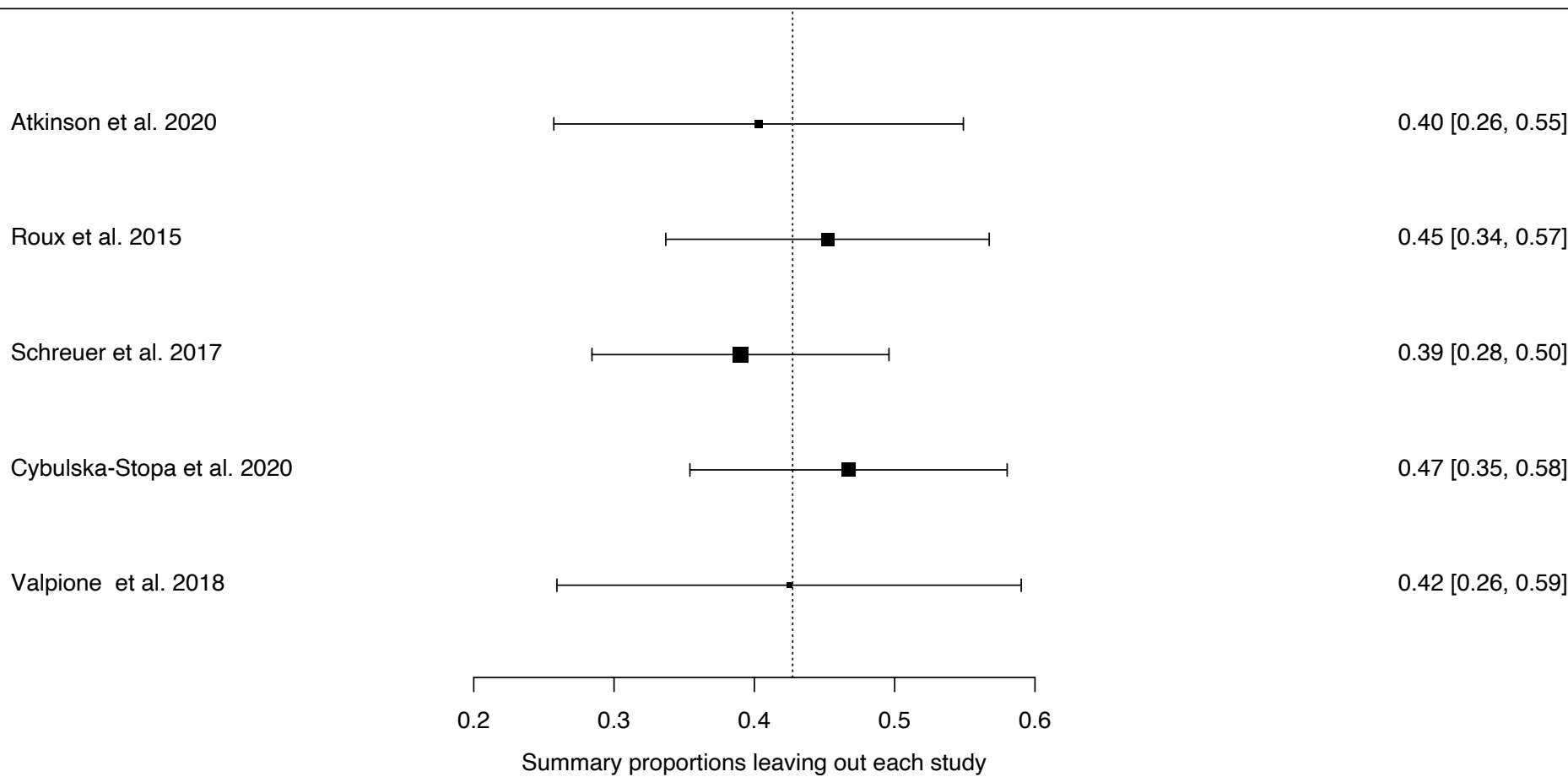

Supplement: Supplementary file 1 [file cancers-15-03754-s001.zip › Supplementary Materials/Figure_S5.pdf]

**A**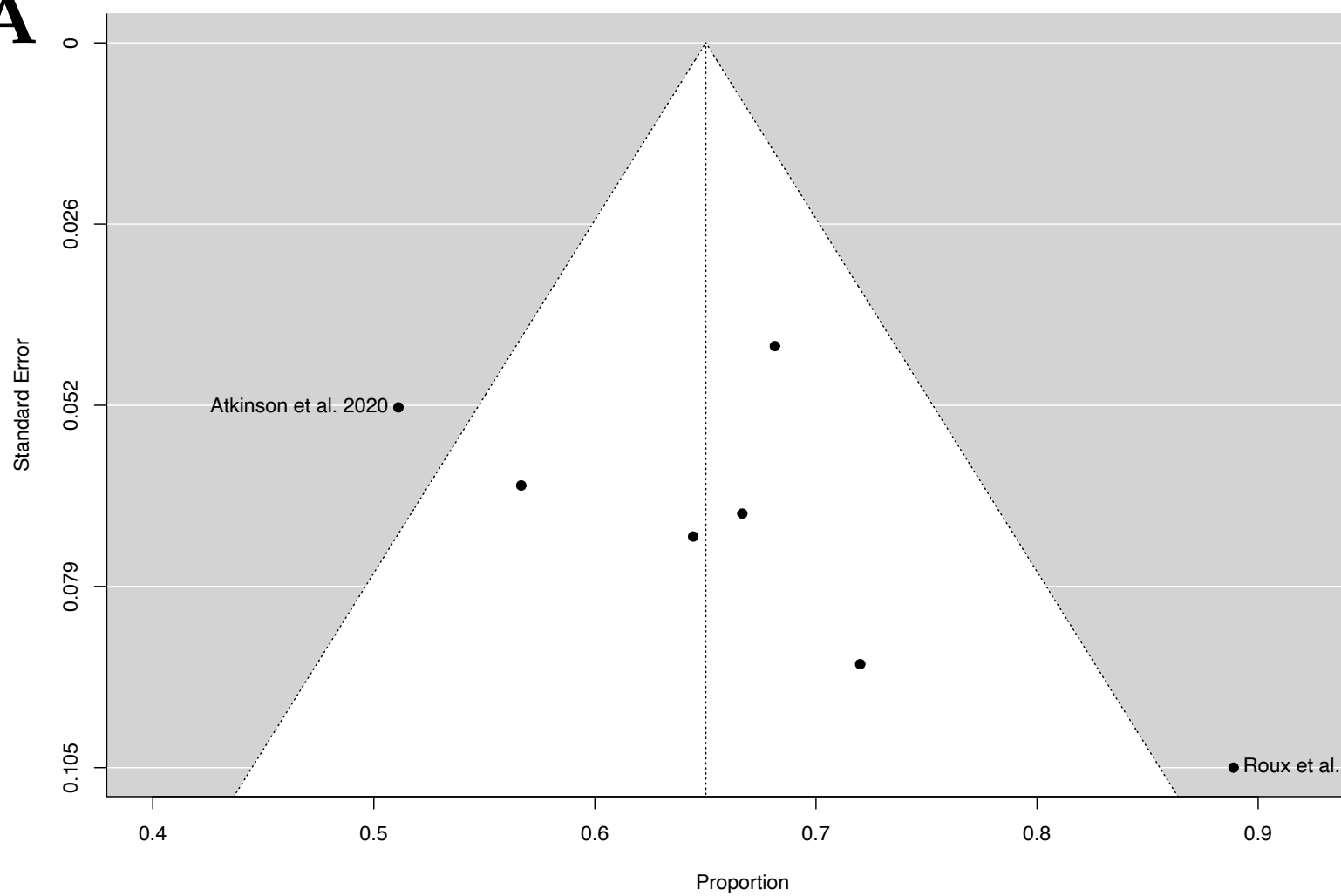**B**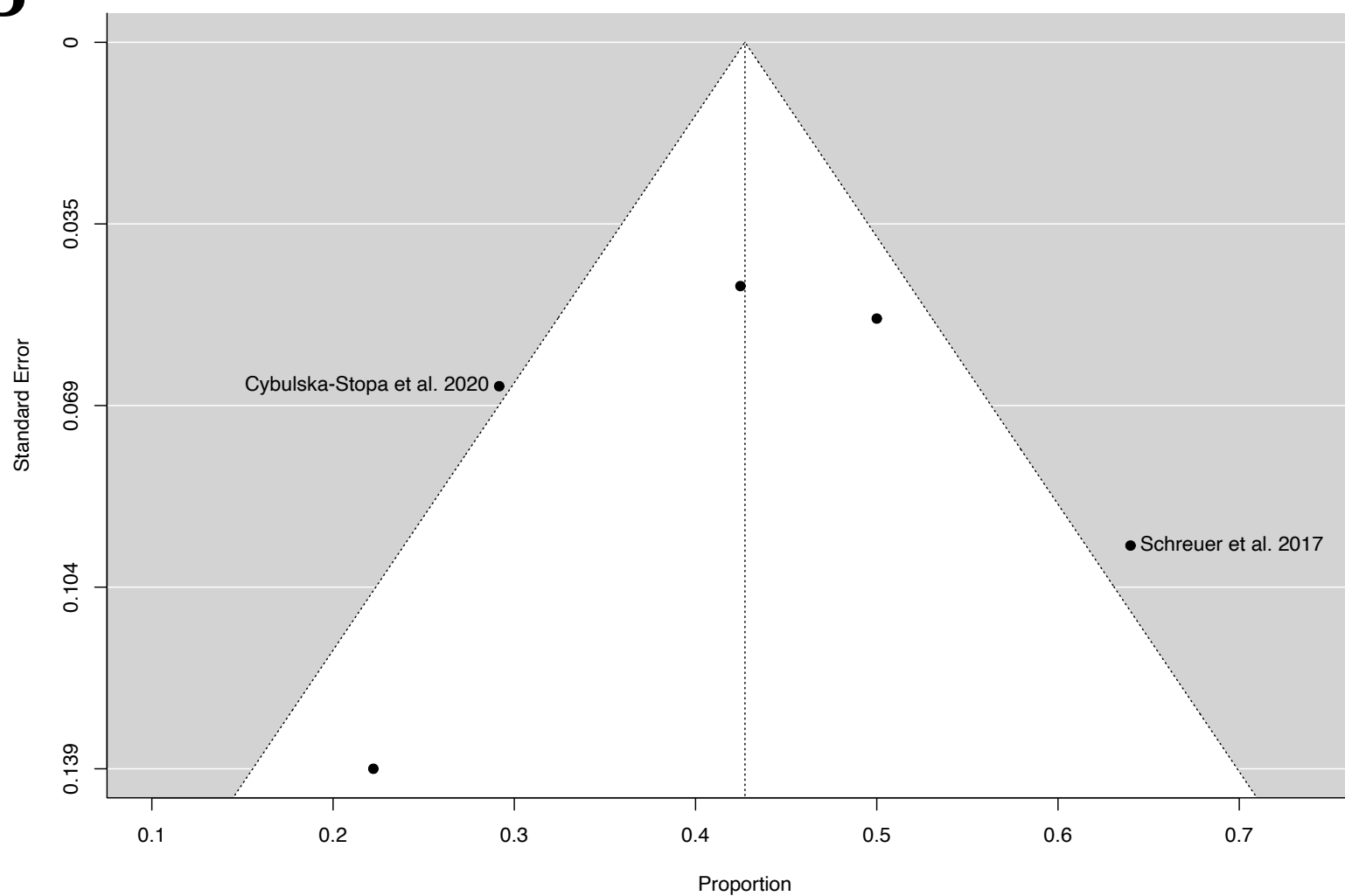

Supplement: Supplementary file 1 [file cancers-15-03754-s001.zip › Supplementary Materials/Figure_S4.pdf]
